# Infection of microglia with *Porphyromonas gingivalis* promotes cell migration and an inflammatory response through the gingipain-mediated activation of protease-activated receptor-2 in mice

**DOI:** 10.1038/s41598-017-12173-1

**Published:** 2017-09-18

**Authors:** Yicong Liu, Zhou Wu, Yurika Nakanishi, Junjun Ni, Yoshinori Hayashi, Fumiko Takayama, Yanmin Zhou, Tomoko Kadowaki, Hiroshi Nakanishi

**Affiliations:** 1 Department of Aging Science and Pharmacology, Faculty of Dental Sciences, Kyushu University, Fukuoka, 812-8582 Japan; 2 OBT Research Center, Faculty of Dental Sciences, Kyushu University, Fukuoka, 812-8582 Japan; 3 Department of Implantology, School of Stomatology, Jilin University, Changchun, 130021 China; 4 Division of Frontier Life Science, Department of Medical and Dental Sciences, Graduate School of Biomedical Sciences, Nagasaki University, Nagasaki, 852-8588 Japan

## Abstract

Despite a clear correlation between periodontitis and cognitive decline in Alzheimer’s disease, the precise mechanism underlying the relationship remains unclear. The periodontal pathogen *Porphyromonas gingivalis* produces a unique class of cysteine proteinases termed gingipains that comprises Arg-gingipain (Rgp) and Lys-gingipain (Kgp). Rgp and Kgp are important in the bacterial mediated host cell responses and the subsequent intracellular signaling in infected cells. In the present study, we attempted to clarify the potential effects of Rgp and Kgp on the cellular activation of brain-resident microglia. We provide the first evidence that Rgp and Kgp cooperatively contribute to the *P*. *gingivalis*-induced cell migration and expression of proinflammatory mediators through the activation of protease-activated receptor 2. The subsequent activation of phosphoinositide 3-kinase/Akt and mitogen-activated protein kinase/extracellular signal-regulated kinase (ERK) kinase/ERK pathways contributes to cell migration and inflammatory response of microglia.

## Introduction

There is increasing evidence that infection of the brain with microbes is linked with Alzheimer’s disease (AD)^1^. Two studies using human brain tissue explored the impact of periodontal infection on AD^2,3^ by examining AD brain tissue specimens using molecular profiling methodologies to identify seven *Treponema* species^2^ as well as the immunogenic endotoxin lipopolysaccharide (LPS) from *Porphyromonas gingivalis*^3^. Furthermore, *P*. *gingivalis* genomic DNA was detected in *Apo E*^−/−^ mouse brains after oral infection^4^. More recently, chronic systemic exposure to LPS from *P*. *gingivalis* has been reported to induce AD-like phenotypes, including microglia-mediated neuroinflammation, intracellular amyloid β (Aβ) accumulation and the impairment of learning and memory functions, in middle-aged mice^5^. In addition to oral pathogens, viruses such as herpes complex virus Type 1^6^ and diverse bacteria, including *Chlamydia pneumonia*^7^ and *Borrelia burgdorferi*^8^, have been found in the brains of patients with AD. Fungal material has also been detected both intra- and extracellularly in the brain of AD patients using specific antibodies against several fungal cells^9^. A recent study in experimental animals showed that the antimicrobial peptide Aβ is involved in combating bacterial and fungal infections^10^. Following these findings, two research groups showed that the elimination of microglia by treatment with colony-stimulating factor 1 receptor kinase inhibitors significantly improved learning ability without affecting the deposition of Aβ in AD model mice^11,12^. These results suggest that neuroinflammation mediated by microglia is a key driver of AD pathology rather than a result of the disease.

A correlation between periodontitis and cognitive decline in AD patients has been reported^13^. However, the precise mechanism underlying the relationship still remains unclear. One possible mechanism is that infection of microbes, including *P*. *gingivalis* in the brain contributes to the chronic neuroinflammation in AD patients via the continuous activation of microglia. *P*. *gingivalis* produces several virulence factors, including outer membrane vesicles, adhesions, LPS, hemolysins and proteases. We have previously reported that LPS derived from *P*. *gingivalis* activates microglia to produce proinflammatory mediators through toll-like 2 receptors^14^. More recently, we reported that UDP-P2Y_6_ receptor system is involved in the microglial process extension to infecting *P*. *gingivalis*^15^. In addition, *P*. *gingivalis* produces a unique class of cysteine proteases termed gingipains that comprises Arg-gingipain (Rgp) and Lys-gingipain (Kgp) in both secretory and cell-associated forms^16,17^. Rgp and Kgp are major factors involved in the bacterial mediated host cell responses and the subsequent intracellular signaling in the infected cells^18^. Therefore, we suggest that Rgp and Kgp are involved in the cellular activation of microglia after infection in the brain, although no information regarding their effects on microglia is available at present. This study attempted to clarify the potential effects of Rgp and Kgp on the cellular activation of microglia.

## Results

### Infection of the brain with *P*. *gingivalis* promotes microglial migration through gingipains

**Figure 1:**
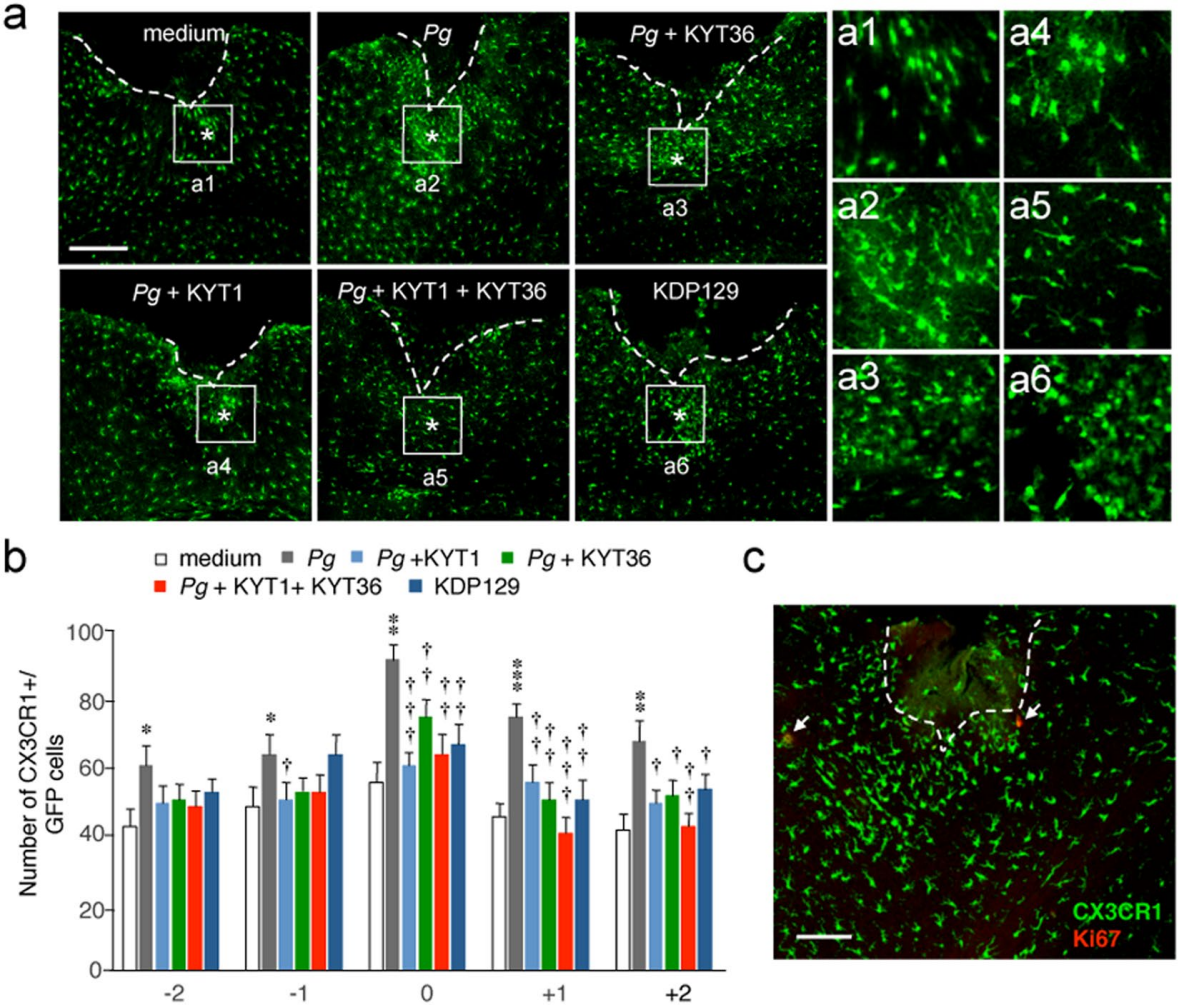
Infection of the *CX3CR1*^+/*GFP*^ mouse brain with *P*. *gingivalis* promotes microglial migration through gingipains. (**a**) CLSM images of the CX3CR1-positive cells accumulated around the injection site (asterisks) of the somatosensory cortex at 24 h after infection. *Pg*: *P*. *gingivalis*. Boxes indicate the 300 × 300 μm squares placed for the cell counting. Scale bar, 300 μm. Asterisks indicate the injection sites (0). **a1**–**a6**: Higher-power micrographs showing most CX3CR1-positive cells that accumulated around the injected sites had a process-bearing morphology. (**b**) The quantitative analyses of CX3CR1-positive cells accumulated around the injection site. The numbers (-2, -1, 0, 1 and 2) represent subsequent slices with reference to the injection site (0). The results represent the mean ± SEM of three independent experiments. A one-way ANOVA with post hoc Tukey’s test; *p* value of medium vs. *Pg*, *Pg* vs. *Pg* + KYT1, *Pg* vs. *Pg* + KYT36, *Pg* vs. *Pg* + KYT1 + KYT36, *Pg* vs. KDP129 were as follows: -2 group: ^*^p = 0.0133, *p* = 0.1140, *p* = 0.1713, *p* = 0.0748, *p* = 0.3583. -1 group: ^*^p = 0.0323, ^†^*p* = 0.0428, *p* = 0.1941, *p* = 0.1941, *p* = 0.9999. 0 group: **p = 0.012, ^†††^*p* = 0.0009, ^††^*p* = 0.0091, ^††^*p* = 0.0022, ^††^*p* = 0.0036. + 1 group: ^***^p = 0.0001, ^††^*p* = 0.0068, ^††^*p* = 0.0029, ^†††^*p* = 0.0006, ^††^*p* = 0.0032. + 2 group: **p = 0.0038, ^†^*p* = 0.0124, ^†^*p* = 0.0194, ^††^*p* = 0.0039, ^†^*p* = 0.0327. (**c**) Immunofluorescent CLSM images of CX3CR1-positive cells devoid of Ki67 immunoreactivity. Scale bar, 100 μm.

To determine whether or not gingipains can promote microglia migration *in vivo*, the microglial accumulation was examined after the injection of live *P*. *gingivalis* into the somatosensory cortex of mice with or without gingipain inhibitors. In order to exclude invaded macrophages, *CX3CR1*^+/*GFP*^ mice were used to count the number of accumulated brain-resident microglia at the site of injection of *P*. *gingivalis*, as CX3CR1 is specifically expressed in microglia^19^. The mean number of GFP^+^ microglia that accumulated around the injection site of *P*. *gingivalis* was significantly larger than that after injection of sterile growth medium (Fig. 1a,b). Next, the potential involvement of gingipains secreted from *P*. *gingivalis* in the cell migration of microglia was examined, as gingipains are associated with the bacterium-mediated host cell responses and the subsequent intracellular signaling in the infected cells. When *P*. *gingivalis* was injected into the somatosensory cortex with either Rgp inhibitor KYT1 or Kgp inhibitor KYT36, the mean number of microglia that accumulated around the injection site of *P*. *gingivalis* was significantly reduced (Fig. 1a,b). Furthermore, the mean number of microglia that accumulated around the injection site of Lys-gingipain mutant *P*. *gingivalis* KDP129 (deletion mutant) which fails to express Kgp were significantly smaller than that after injection of *P*. *gingivalis* (Fig. 1a,b). To examine a possible involvement of cell proliferation, immunostaining using anti-Ki67 antibody was performed. The process-bearing bright CX3CR1-positive cells accumulated around the injection site were negative for Ki67, a crucial cellular proliferation marker (Fig. 1c), suggesting that microglial migration is at the basis of *P*. *gingivalis*-induced microgliosis.

### Infection of microglia with *P*. *gingivalis* induces cell migration through gingipains

**Figure 2:**
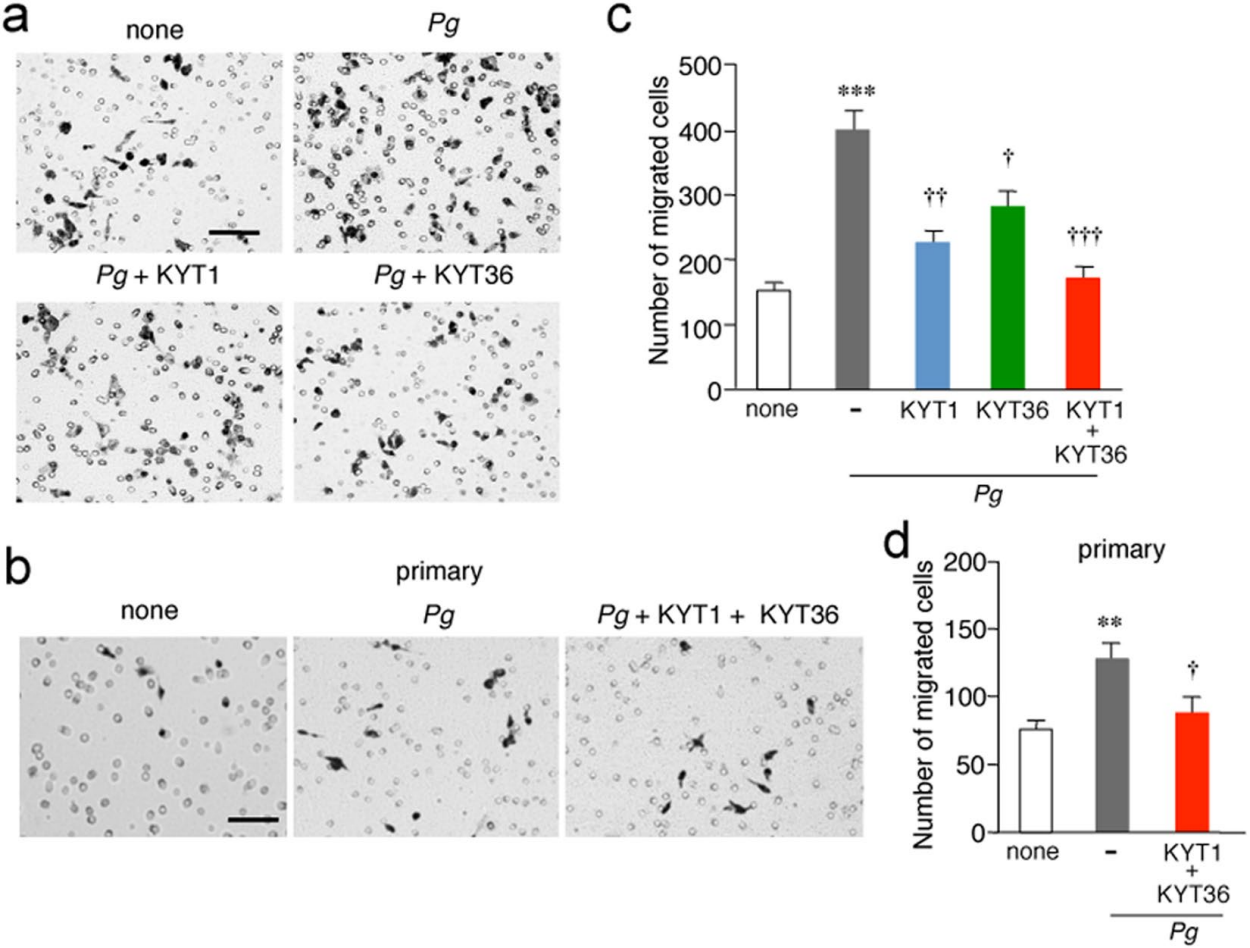
Gingipains promote the cell migration after the infection of microglia with *P*. *gingivalis*. (**a**,**b**) Representative images of migrated MG6 cells (**a**) and primary cultured microglia (**b**). The Boyden chamber assay was performed to evaluate the cell migration after infection of MG6 cells and primary cultured microglia with *P*. *gingivalis* in the presence or absence of KYT1 (1 μM) and KYT36 (1 μM). MG6 cells and primary cultured microglia migrated through a membrane were stained and counted after 12 h. Scale bar, 100 μm. (**c**, **d**) The quantitative analyses of the number of migrated MG6 cells (**c**) and primary cultured microglia (**d**). The results represent the mean ± SEM of three independent experiments. A one-way ANOVA with post hoc Tukey’s test; (**c**) none vs. *Pg*: ***p = 0.0001, *Pg* vs. *Pg* + KYT1: ^††^*p* = 0.0015, *Pg* vs. *Pg* + KYT36: ^†^*p* = 0.0239, *Pg* vs. *Pg* + KYT1 + KYT36: ^†††^*p* = 0.0002; (**d**) none vs. *Pg*: **p = 0.0059, *Pg* vs. *Pg* + KYT1 + KYT36: ^†^*p* = 0.0187.

To further address that microglial accumulation around the injection site of *P*. *gingivalis* in the somatosensory cortex is due to cell migration but not to cell proliferation, *in vitro* migration assay using a Boyden chamber was performed. *P*. *gingivalis* infection induced the cell migration of MG6 cells (Fig. 2a) and primary cultured microglia (Fig. 2b) through the polycarbonate membrane to a greater degree than seen in untreated controls. The potential involvement of extracellular nucleotides, including ADP or ATP, in the cell migration of microglia was next examined, as cortical microglia extended their processes towards focally injected *P*. *gingivalis* through the UDP-P2Y_6_ receptor system^15^. However, neither MRS2578, a selective inhibitor of P2Y_6_ receptors, nor PSB0739, a selective inhibitor of P2Y_12_ receptors, had any effect on the *P*. *gingivalis* infection-induced MG6 cell migration (Supplementary Fig. S1). KYT1 and KYT36 significantly inhibited the *P*. *gingivalis* infection-induced cell migration of MG6 cells when administered separately, and their combined administration almost completely inhibited the migration (Fig. 2a,c). A combined administration of KYT1 and KYT36 also significantly inhibited the *P*. *gingivalis* infection-induced cell migration of primary cultured microglia (Fig. 2b,d). On the other hand, *P*. *gingivalis* LPS failed to induce cell migration of MG6 cells, whereas *E*. *coli* LPS induced a significant cell migration of MG6 cells (Supplementary Fig. S1).

### Gingipains induce the proteolytic activation of protease-activated receptor (PAR) 2 in microglia after infection with *P*. *gingivalis*

**Figure 3:**
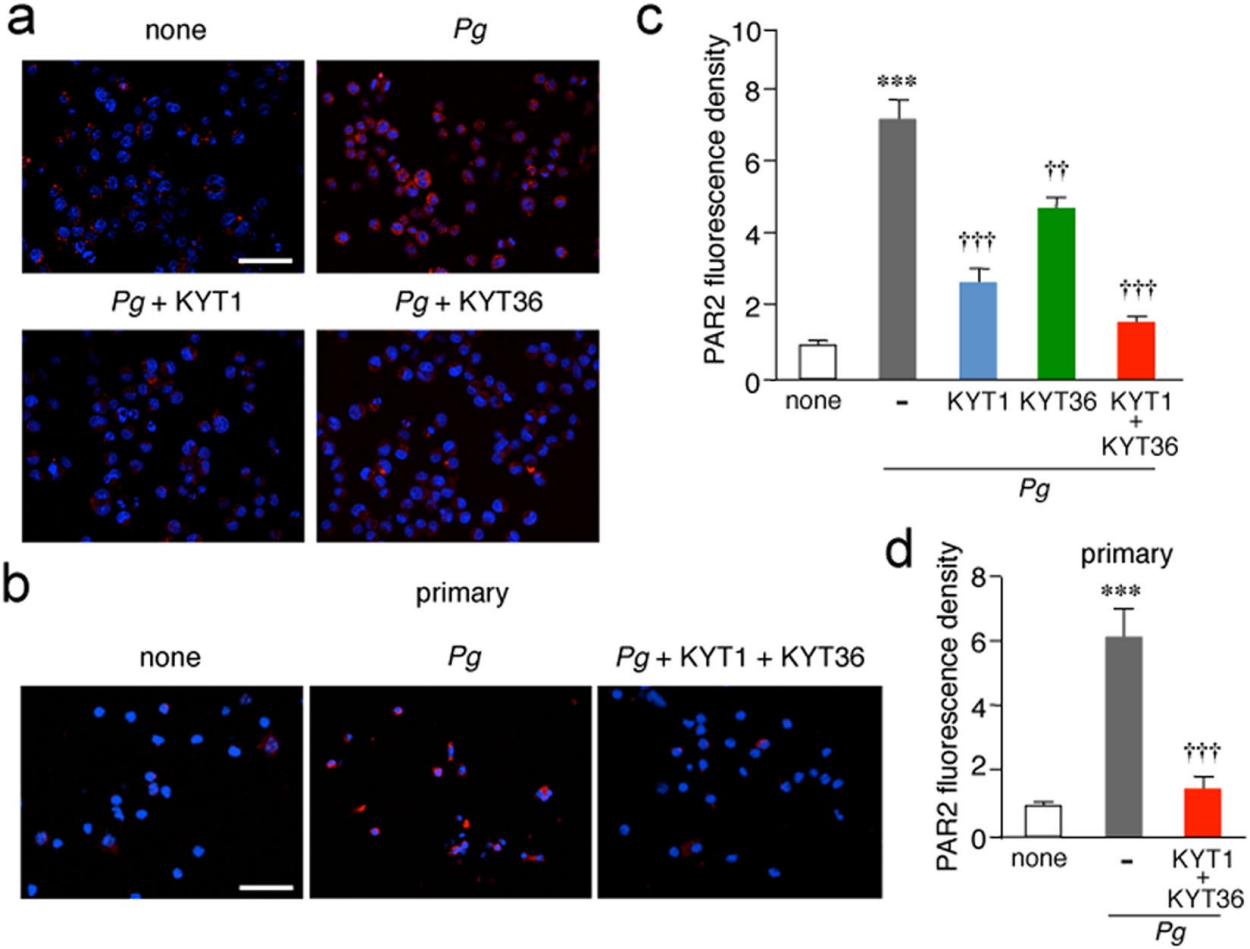
Gingipains promote the proteolytic activation of PAR2 after the infection of microglia with *P*. *gingivalis*. (**a**,**b**) Immunofluorescent CLMS images of cleaved PAR2 after infection of MG6 cells (**a**) and primary cultured microglia (**b**) with *P*. *gingivalis*. MG6 cells and primary cultured microglia were infected with *P*.*gingivalis* for 12 h in the presence or absence of KYT1 (1 μM) and KYT36 (1 μM) and then stained with anti-PAR2 antibody (S-19, red) followed by nuclear staining with Hoechst (blue). Scale bar, 100 μm. (**c**,**d**) The quantitative analyses of the intensity of immunofluorescence for cleaved PAR2 in MG6 cells (**c**) and primary cultured microglia (**d**). The results represent the mean ± SEM of three independent experiments. A one-way ANOVA with post hoc Tukey’s test; (**c**) none vs. *Pg*: ***p = 0.0001, *Pg* vs. *Pg* + KYT1: ^†††^*p* = 0.0001, *Pg* vs. *Pg* + KYT36: ^††^*p* = 0.0021, *Pg* vs. *Pg* + KYT1 + KYT36: ^†††^*p* = 0.0001; (**d**) none vs. *Pg*: ***p = 0.0004, *Pg* vs. *Pg* + KYT1 + KYT36: ^†††^*p* = 0.0007.

The four PAR family members (PAR1, 2, 3 and 4) are G-protein-coupled receptors with a unique mechanism of activation. These receptors carry their own tethered ligands and are activated by serine proteases such as trypsin and tryptase. PARs in epithelial cells is also activated by trypsin-like cysteine proteases and gingipains^20–22^. The effects of infection with *P*. *gingivalis* on the activation of PAR2 in MG6 cells was examined, as human microglia only express PAR2^23^. S-19, which specifically recognizes the activated form of PAR2, was used to examine the effect of *P*. *gingivalis* infection on the activation of PAR2 expressed on MG6 cells and primary cultured microglia. Infection with *P*. *gingivalis* significantly increased the levels of the activated form of PAR2 in MG6 cells (Fig. 3a) and primary cultured microglia (Fig. 3b). KYT1 and KYT36 significantly inhibited the PAR2 activation when administered separately, and their combined administration almost completely inhibited the *P*. *gingivalis* infection-induced activation of PAR2 in MG6 cells (Fig. 3a,c). A combined administration of KYT1 and KYT36 also significantly inhibited the *P*. *gingivalis* infection-induced activation of PAR2 in primary cultured microglia (Fig. 3b,d).

### PAR2 activation and subsequent phosphorylation of Akt and ERK1/2 are involved in the *P*. *gingivalis* infection-induced microglial migration

**Figure 4:**
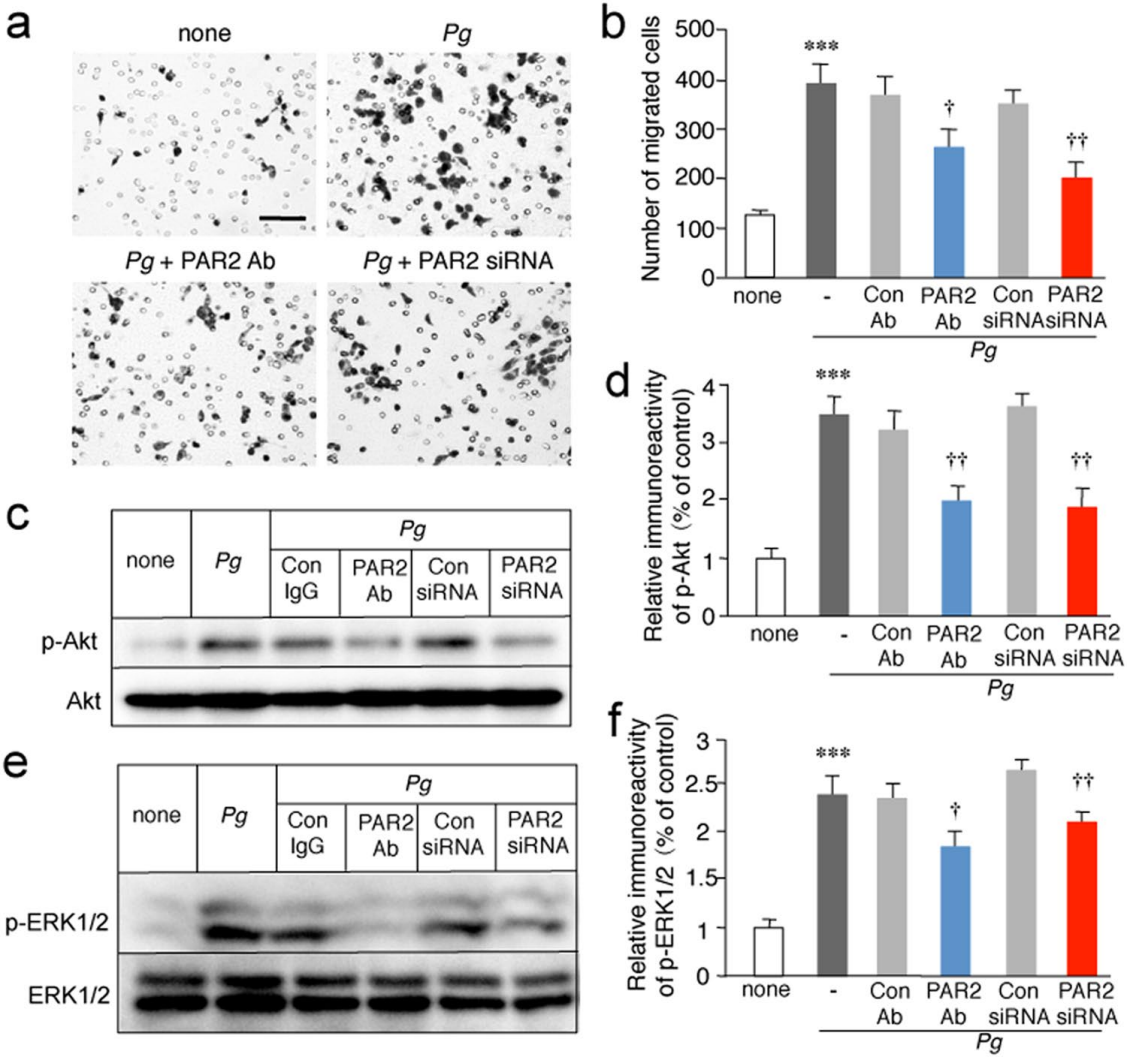
Involvement of PAR2 activation and subsequent phosphorylation of Akt and ERK1/2 in the *P*. *gingivalis* infection-induced microglial migration. (**a**) Representative images of migrated MG6 cells. The Boyden chamber assay was performed to evaluate cell migration after infection of MG6 cells with *P*. *gingivalis* in the presence or absence of control Ab (1 μg/ml), PAR2 neutralization antibody (SAM11; 1 μg/ml), control siRNA (50 nM) and PAR2 siRNA (50 nM). Microglial cells that migrated through a membrane were stained and counted after 12 h. Scale bar, 100 μm. (**b**) The quantitative analyses of the number of migrated cells. The results represent the mean ± SEM of three independent experiments. A one-way ANOVA with post hoc Tukey’s test; none vs. *Pg*: ***p = 0.0004, *Pg* + Con Ab vs. *Pg* + PAR2 Ab: ^†^*p* = 0.0328, *Pg* + Con siRNA vs. *Pg* + PAR2 siRNA: ^††^*p* = 0.0036. (**c**) The immunoblots show p-Akt and total Akt after infection of MG6 cells with *P*. *gingivalis* in the presence or absence of PAR2 neutralization antibody (1 μg/ml) or PAR2 siRNA (50 nM) at 2 h. (**d**) The quantitative analyses of the p-Akt were shown. The results represent the mean ± SEM of three independent experiments. A one-way ANOVA with post hoc Tukey’s test; none vs. *Pg*: ***p = 0.0001, *Pg* + Con Ab vs. *Pg* + PAR2 Ab: ^††^*p* = 0.0027, *Pg* + Con siRNA vs. *Pg* + PAR2 siRNA: ^††^*p* = 0.0021. (**e**) The immunoblots show p-ERK1/2 and total ERK1/2 after infection of MG6 cells with *P*. *gingivalis* in the presence or absence of PAR2 neutralization antibody (1 μg/ml) or PAR2 siRNA (50 nM) at 30 min. (**f**) The quantitative analyses of the p-ERK were shown. The results represent the mean ± SEM of three independent experiments. A one-way ANOVA with post hoc Tukey’s test; none vs. *Pg*: ***p = 0.0001, *Pg* + Con Ab vs. *Pg* + PAR2 Ab: ^†^*p* = 0.0103, *Pg* + Con siRNA vs. *Pg* + PAR2 siRNA: ^††^*p* = 0.0074. Full bots are presented in Supplementary Fig. S6.

Next, the potential involvement of PAR2 in the *P*. *gingivalis*-induced cell migration of MG6 cells was examined. The *P*. *gingivalis* infection-induced cell migration of MG6 cells was significantly inhibited by the administration of the PAR2 neutralization antibody SAM11 and by PAR2 siRNA. In contrast, the control antibody (Ab) and siRNA had no effect (Fig. 4a,b).

The signaling pathways that are triggered by PAR2 activation may activate several downstream intracellular signaling pathways, including phosphoinositide 3-kinase (PI3K)/Akt^24^ and mitogen-activated protein kinase/extracellular signal-regulated kinase (ERK) kinase (MEK)/ERK pathways^25^, which are responsible for a variety of microglial cellular activities including cell migration^26^. We then examined the potential activation of PI3K/Akt and MEK/ERK pathways after the infection of MG6 cells and primary cultured microglia with *P*. *gingivalis*. Akt is a serine/threonine protein kinase activated by stimuli that induce the production of phosphatidylinositol (3,4,5)-trisphosphate through activation of PI3K^27^. Akt and ERK1/2 were phosphorylated after the infection of MG6 cells with *P*. *gingivalis* (Supplementary Fig. S2). Blockade of PAR2-mediated intracellular signaling using the neutralizing antibody SAM11 or siRNA significantly inhibited the *P*. *gingivalis* infection-induced phosphorylation of Akt and ERK1/2 in MG6 cells (Fig. 4c,d) and primary cultured microglia (Fig. 4e,f). In contrast, control Ab and siRNA had no effect.

### Infection of microglia with *P*. *gingivalis* promotes cell migration and membrane ruffling through the activation of the PI3K/Akt and MEK/ERK pathways

**Figure 5:**
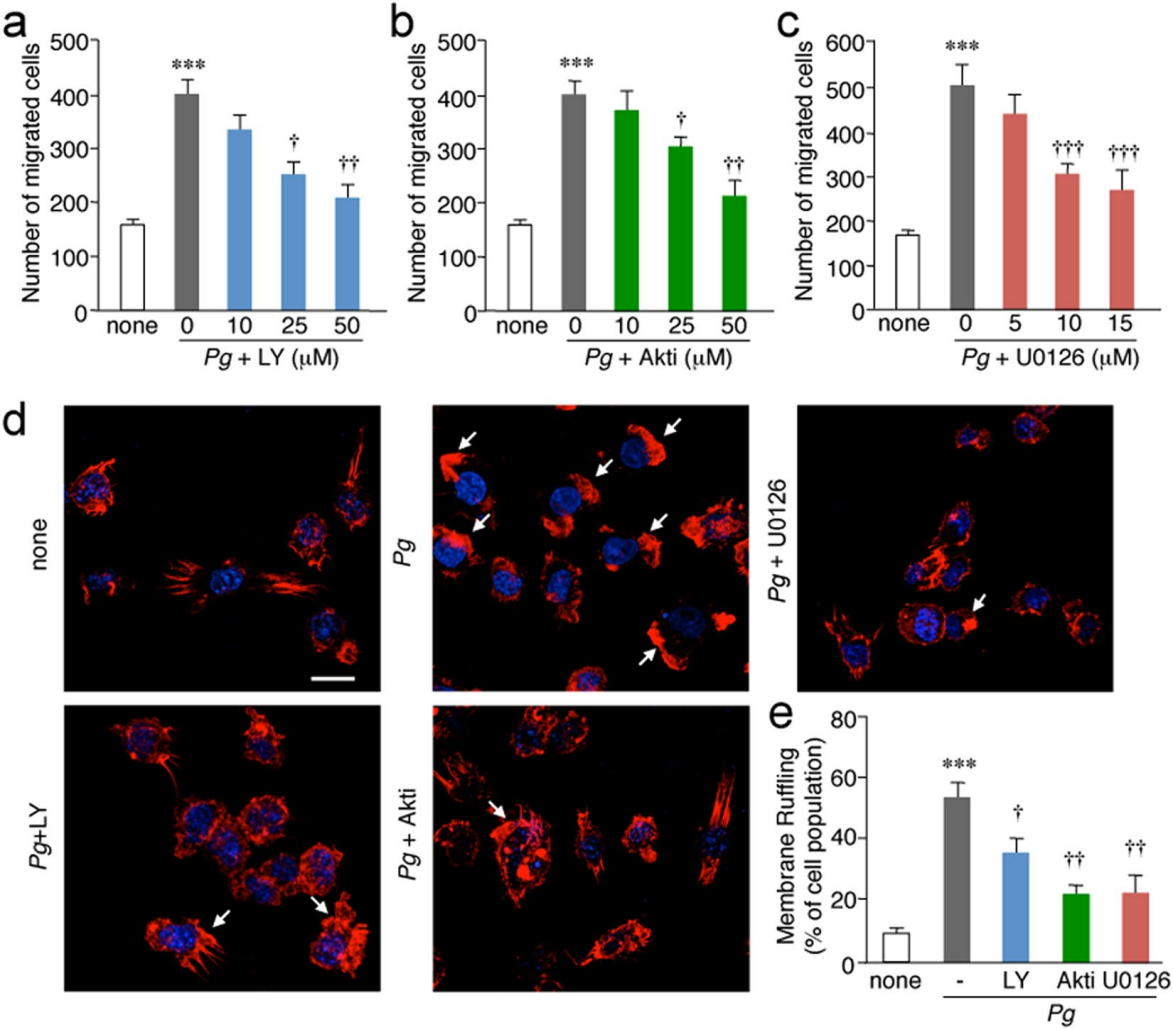
Involvement of the PI3K/Akt and MEK/ERK pathways in the cell migration and membrane ruffling formation after the infection of microglia with *P*. *gingivalis*. (**a**,**b**,**c**) The quantitative analyses of the number of cells migrated after the infection of MG6 cells with *P*. *gingivalis* in the absence or presence of LY294002 (LY, **a**), Akti (**b**) or U0126 (**c**) at three different concentrations. MG6 cells that migrated through a membrane were stained and counted after 12 h. The results represent the mean ± SEM of three independent experiments. A one-way ANOVA with post hoc Tukey’s test; LY294002 group: none vs. *Pg*: ^***^p = 0.0007, *Pg* vs. *Pg* + 10 μM: *p* = 0.4615, *Pg* vs. *Pg* + 25 μM: ^†^*p* = 0.0261, *Pg* vs. *Pg* + 50 μM: ^††^*p* = 0.0041. Akti group: none vs. *Pg*: ***p = 0.0005, *Pg* vs. *Pg* + 10 μM: *p* = 0.9008, *Pg* vs. *Pg* + 25 μM: ^†^*p* = 0.0486, *Pg* vs. *Pg* + 50 μM: ^††^*p* = 0.0033. U0126 group: none vs. *Pg*: ^***^p = 0.0001, *Pg* vs. *Pg* + 5 μM: *p* = 0.1123, *Pg* vs. *Pg* + 10 μM: ^†††^*p* = 0.0001, *Pg* vs. *Pg* + 15 μM: ^†††^*p* = 0.0001. (**d**) CLSM images of F-actin after infection of MG6 cells with *P*. *gingivalis* in the presence or absence of LY294002 (LY, 50 μM), Akti (50 μM) and U0126 (15 μM). At 2 h after infection of MG6 cells with *P*. *gingivalis*, MG6 cells were stained with Texas Red-X Phalloidin (red) and Hoechst (blue) for 1 h. Arrows indicate membrane ruffles. Scale bar, 5 μm. (**e**) The quantitative analyses of the membrane ruffling after the infection of MG6 cells with *P*. *gingivalis*. The mean relative population of MG6 cells showing membrane ruffling was counted, and the results represent the mean ± SEM of three independent experiments. A one-way ANOVA with post hoc Tukey’s test; none vs. *Pg*: ***p = 0.0002, *Pg* vs. *Pg* + LY: ^†^*p* = 0.0479, *Pg* vs. *Pg* + Akti: ^††^*p* = 0.0021, *Pg* vs. *Pg* + U0126: ^††^*p* = 0.0031.

Both the PI3K inhibitor LY294002 and the Akt inhibitor Akti significantly inhibited the *P*. *gingivalis* infection-induced cell migration of MG6 cells in a dose-dependent manner (Fig. 5a,b). Furthermore, ERK inhibition with U0126 also significantly inhibited the *P*. *gingivalis* infection-induced cell migration of MG6 cells in a dose-dependent manner (Fig. 5c). MG6 cells were stained with phalloidin to visualize actin polymerization after *P*. *gingivalis* infection, as actin polymerization and membrane ruffling are essential steps for cell migration. After the infection of MG6 cells with *P*. *gingivalis*, the F-actin of MG6 cells was polymerized to induce membrane ruffling (Fig. 5d arrows). Regarding the mechanism of membrane ruffling after the infection of MG6 cells with *P*. *gingivalis*, the possible involvement of the PI3K/Akt and MEK/ERK pathways was examined. The treatment with LY294002, Akti and U0126 significantly inhibited the *P*. *gingivalis* infection-induced membrane ruffling of MG6 cells (Fig. 5d,e).

### Infection of microglia with *P*. *gingivalis* promotes expression of proinflammatory mediators through activation of PAR2 by gingipains

**Figure 6:**
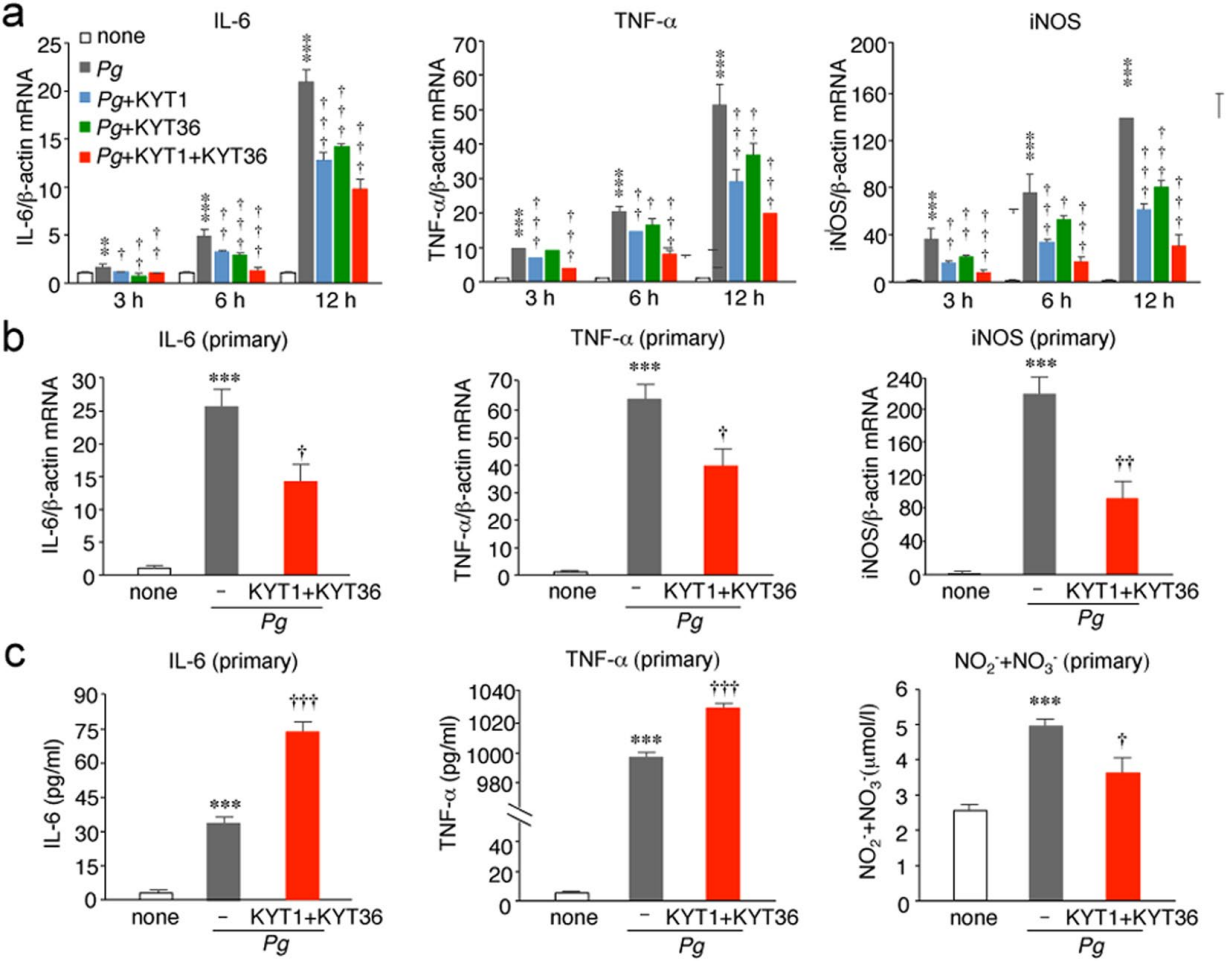
The involvement of gingipains in the *P*. *gingivalis* infection–induced expression of proinflammatory mediators in microglia. (**a**) The quantitative analyses of the mRNA expression of IL-6, TNF-α and iNOS after the infection of MG6 cells with *P*. *gingivalis* in the presence and absence of KYT1(1 μM) and KYT36 (1 μM) at each time point. The results represent the mean ± SEM of three independent experiments. A one-way ANOVA with post hoc Tukey’s test; none vs. *Pg*, *Pg* vs. *Pg* + KYT1, *Pg* vs. *Pg* + KYT36, *Pg* vs. *P*.*g* + KYT1 + KYT36 were as follows: IL-6 group: 3 h, ^**^p = 0.0041, ^†^*p* = 0.0172, ^††^*p* = 0.001, ^††^*p* = 0.0048; 6 h, ^***^p = 0.0001, ^††^*p* = 0.0017,^†††^*p* = 0.0004, ^†††^*p* = 0.0001; 12 h, ^***^p = 0.0001, ^†††^*p* = 0.0001, ^†††^*p* = 0.0001, ^†††^*p* = 0.0001. TNF-α group: 3 h, ^***^p = 0.0001, ^†††^*p* = 0.0001, *p* = 0.3286, ^†††^*p* = 0.0001; 6 h, ^***^p = 0.0001, ^††^*p* = 0.0013, ^†^*p* = 0.0193, ^†††^*p* = 0.0001; 12 h, ^***^p = 0.0001, ^†††^*p* = 0.0001, ^††^*p* = 0.0036, ^†††^*p* = 0.0001. iNOS group: 3 h, ^***^p = 0.0001, ^††^*p* = 0.0012, ^††^*p* = 0.0093, ^†††^*p* = 0.0001; 6 h, ***p = 0.0001, ^†††^*p* = 0.0003, ^†^*p* = 0.0295, ^†††^*p* = 0.0001; 12 h, ***p = 0.0001, ^†††^*p* = 0.0001, ^†††^*p* = 0.0005, ^†††^*p* = 0.0001. (**b**) The quantitative analyses of the mRNA expression of IL-6, TNF-α and iNOS after the infection of primary cultured microglia with *P*. *gingivalis* in the presence and absence of KYT1(1 μM) and KYT36 (1 μM). The results represent the mean ± SEM of three independent experiments. A one-way ANOVA with post hoc Tukey’s test; none vs. *Pg*, *Pg* vs. *P*.*g* + KYT1 + KYT36 were as follows: IL-6 group: ***p = 0.0003, ^†^*p* = 0.0146. TNF-α group: ***p = 0.0001, ^††^*p* = 0.0251. iNOS group: ***p = 0.0001, ^††^*p* = 0.0015. (**c**) The mean amounts of IL-6, TNF-α and NO_2_^−^ + NO_3_^−^ in the culture medium were measured after the infection of primary cultured microglia with *P*. *gingivalis* in the presence and absence of KYT1 (1 μM) and KYT36 (1 μM) for 24 h. The results represent the mean ± SEM of three independent experiments. A one-way ANOVA with post hoc Tukey’s test; none vs. *Pg*, *Pg* vs. *Pg* + KYT1 + KYT36 were as follows: IL-6 group: ^***^p = 0.0001, ^†††^*p* = 0.0001. TNF-α group: ***p = 0.0001, ^†††^*p* = 0.0001. NO_2_^−^ + NO_3_^−^ group: ***p = 0.0009, ^†^*p* = 0.0172.

Infection of MG6 cells with *P*. *gingivalis* significantly increased the mRNA expression levels of proinflammatory mediators, including interleukin-6 (IL-6), tumor necrosis factor-α (TNF-α) and inducible nitric oxide synthase (iNOS) (Fig. 6a), without affecting the mRNA expression of anti-inflammatory mediators, including IL-10, arginase-1 and IL-4 (Supplementary Fig. S3). Both KYT1 and KYT36 significantly inhibited the *P*. *gingivalis* infection-induced expression of IL-6, TNF-α and iNOS in MG6 cells (Fig. 5a). The combined use of KYT1 and KYT36 further inhibited the *P*. *gingivalis* infection-induced expression of IL-6, TNF-α and iNOS in MG6 cells. *P*. *gingivalis* infection also induced expression of IL-6, TNF-α and iNOS in primary cultured microglia. A combined administration of KYT1 and KYT36 significantly inhibited the *P*. *gingivalis* infection-induced expression of IL-6, TNF-α and iNOS in primary cultured microglia (Fig. 6b). Furthermore, the infection of primary cultured microglia with *P*. *gingivalis* also significantly increased the secretion of IL-6 and TNF-α, and the accumulation of NO metabolites (Fig. 6c). A combined administration of KYT1 and KYT36 significantly increased the mean amounts of IL-6 and TNF-α in the culture medium after infection of *P*. *gingivalis*. On the other hand, their combined administration significantly decreased the mean amount of NO metabolites in the culture medium (Fig. 6c).

**Figure 7:**
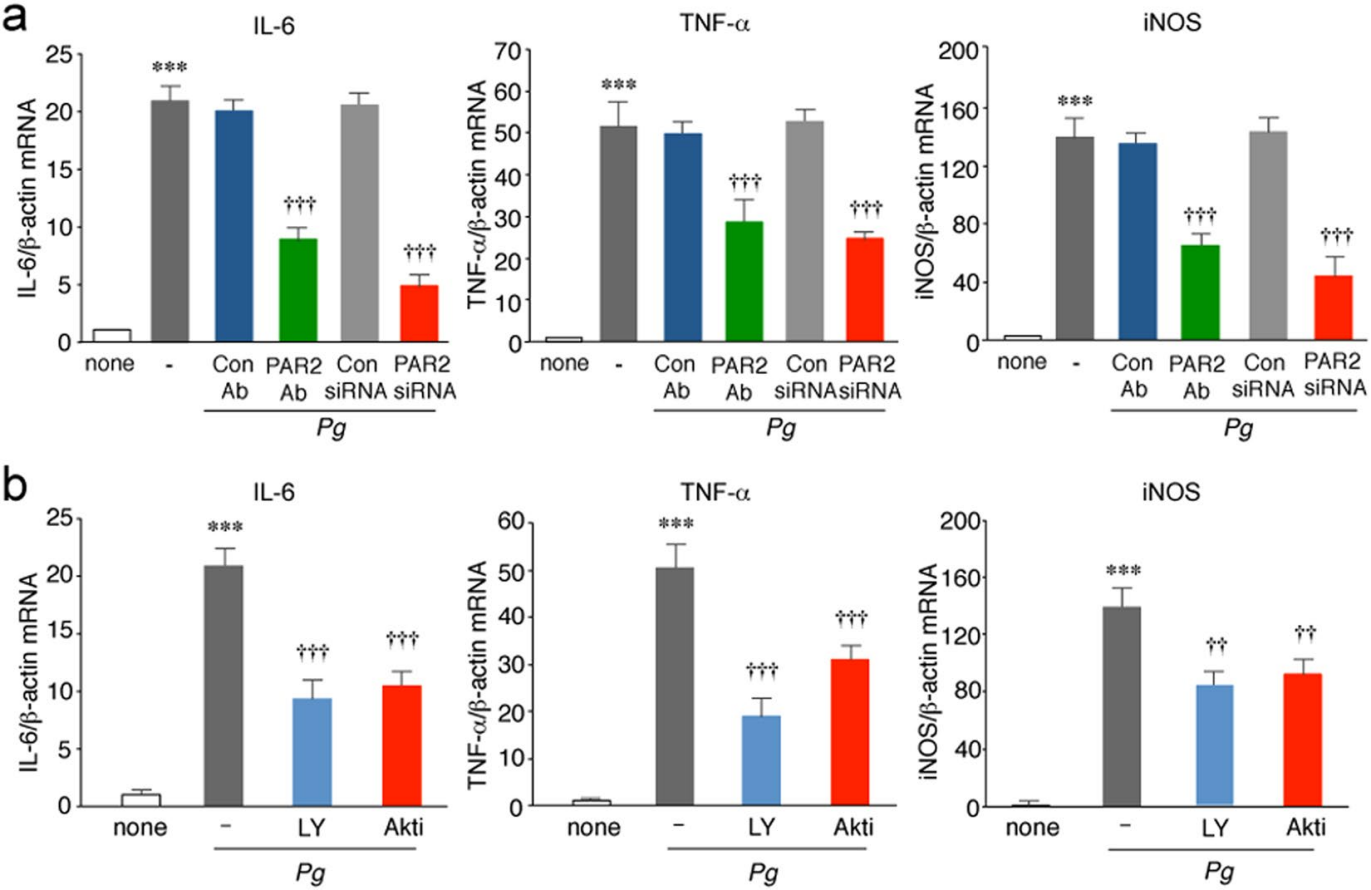
The involvement of PAR2 and PI3K/Akt pathway in the *P*. *gingivalis* infection–induced expression of proinflammatory mediators by microglia. (**a**) The quantitative analyses of the mRNA expression of IL-6, TNF-α and iNOS after the infection of MG6 cells with *P*. *gingivalis* in the presence and absence of PAR2 Ab or PAR2 siRNA at 12 h. The results represent the mean ± SEM of three independent experiments. A one-way ANOVA with post hoc Tukey’s test; none vs. *Pg*, *Pg* + Con Ab vs. *Pg* + PAR2 Ab, *Pg* + Con siRNA vs. *Pg* + PAR2 siRNA were as followed: IL-6 group: ^***^p = 0.0001, ^†††^*p* = 0.0001, ^†††^*p* = 0.0001. TNF-α group: ***p = 0.0001, ^†††^*p* = 0.0007, ^†††^*p* = 0.0002. iNOS group: ^***^p = 0.0001, ^†††^*p* = 0.0002, ^†††^*p* = 0.0001. (**b**) The quantitative analyses of the mRNA expression of IL-6, TNF-α and iNOS after the infection of MG6 cells with *P*. *gingivalis* in the presence and absence of LY294002 (LY, 50 μM) and Akti (50 μM) at 12 h. The results represent the mean ± SEM of three independent experiments. A one-way ANOVA with post hoc Tukey’s test; none vs. *Pg*, *Pg* vs. *Pg* + LY, *Pg* vs. *Pg* + Akti were as follows: IL-6 group: ***p = 0.0001, ^†††^*p* = 0.0001, ^†††^*p* = 0.0001. TNF-α group: ***p = 0.0001, ^†††^*p* = 0.0001, ^†††^*p* = 0.0009. iNOS group: ***p = 0.0001, ^††^*p* = 0.0014, ^††^*p* = 0.0052.

Next, a possible involvement of PAR2 in the *P*. *gingivalis* infection-induced expression of IL-6, TNF-α and iNOS was examined. The PAR2 neutralization antibody SAM11 or PAR2 siRNA significantly inhibited *P*. *gingivalis* infection-induced expression of IL-6, TNF-α and iNOS in MG6 cells (Fig. 7a). In contrast, control Ab and siRNA had no effect. Furthermore, either treatment with LY294002 as well as Akti significantly inhibited *P*. *gingivalis* infection-induced expression of IL-6, TNF-α and iNOS in MG6 cells (Fig. 7b).

### Infection of microglia with *P*. *gingivalis* induce microglia-mediated neuroinflammation through the activation of PAR2 and Toll-like receptor 2 (TLR2)

**Figure 8:**
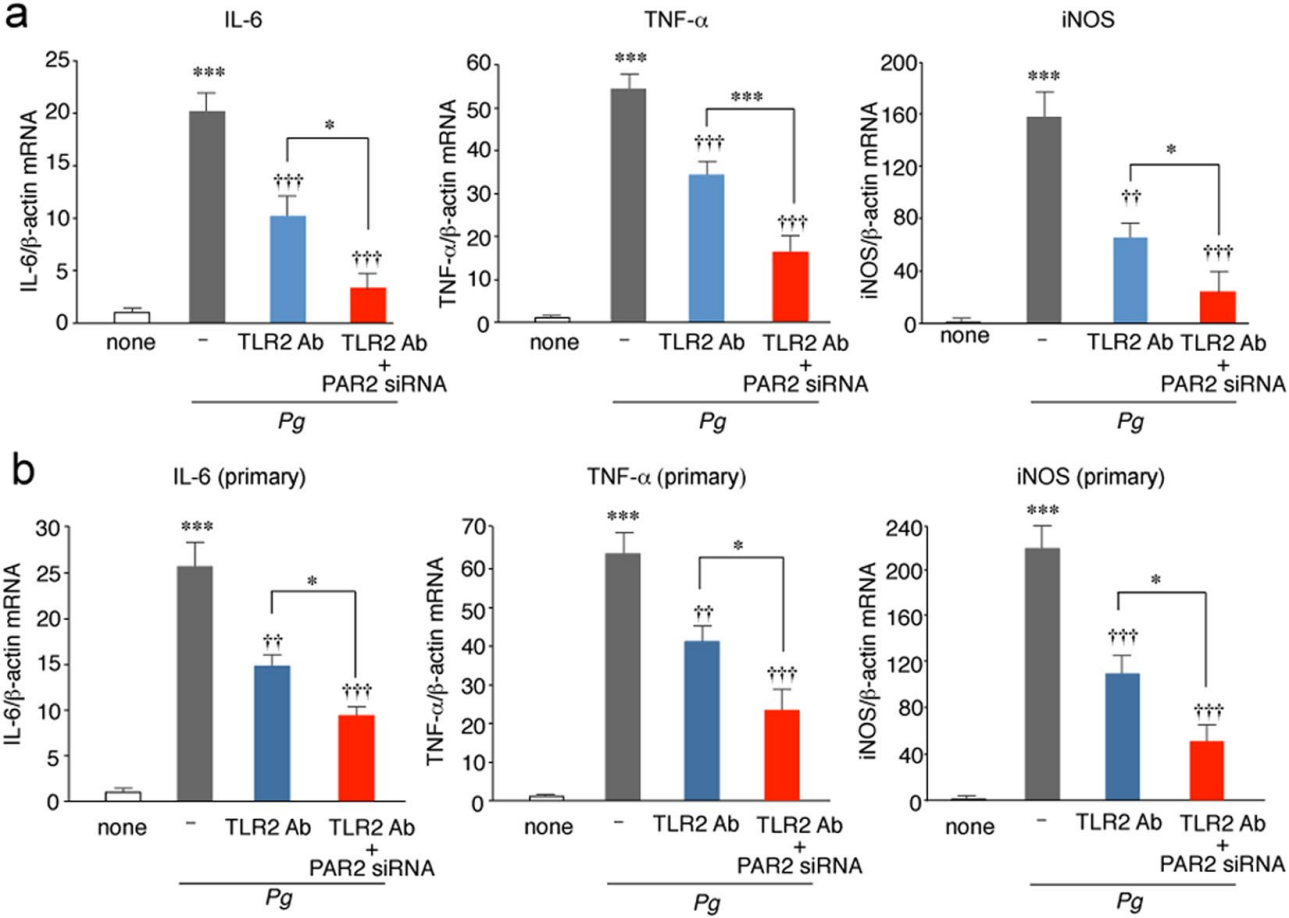
A link between gingipain/PAR2 and LPS from *P*. *gingivalis*/TRL2 pathways in the *P*. *gingivalis* infection-induced expression of inflammatory mediators by microglia. (**a**,**b**) The quantitative analyses of the mRNA expression of IL-6, TNF-α and iNOS after the infection of MG6 cells (**a**) and primary cultured microglia (**b**) with *P*. *gingivalis* in the presence and absence of TLR2 antibody (1 μg/ml) and PAR2 siRNA (50 nM). The results represent the mean ± SEM of three independent experiments. A one-way ANOVA with post hoc Tukey’s test; (**a**) none vs. *Pg*, *Pg* vs. *Pg *+ TLR2 Ab, *Pg* vs. *Pg* + TLR2 Ab + PAR2 siRNA, *Pg* + TLR2 Ab vs. *Pg* + TLR2 Ab + PAR2 siRNA were as follows: IL-6 group: ***p = 0.0001, ^††^*p* = 0.0039, ^†††^*p* = 0.0005, ^*^p = 0.0472. TNF-α group: ***p = 0.0001, ^†††^*p* = 0.0002, ^†††^*p* = 0.0001, ***p = 0.0001. iNOS group: ***p = 0.0001, ^†††^*p* = 0.0004, ^†††^*p* = 0.0001, *p = 0.0474; (**b**) none vs. *Pg*, *Pg* vs. *Pg* + TLR2 Ab, *Pg* vs. *Pg* + TLR2 Ab + PAR2 siRNA, *Pg* + TLR2 Ab vs. *Pg* + TLR2 Ab + PAR2 siRNA were as follows: IL-6 group: ***p = 0.0001, ^††^*p* = 0.0014, ^†††^*p* = 0.0001, *p = 0.0347. TNF-α group: ***p = 0.0001, ^††^*p* = 0.0042, ^†††^*p* = 0.0001, *p = 0.0315. iNOS group: ***p = 0.0001, ^†††^*p* = 0.0006, ^†††^*p* = 0.0001, *p = 0.0237.

Next, we examined a possible crosstalk of PAR2- and TLR2-regulated inflammatory activation of microglia, because *P*. *gingivalis* LPS activates microglia to produce proinflammatory mediators through TLR2^14^. TLR2 Ab significantly inhibited the *P*. *gingivalis* infection-induced expression of IL-6, TNF-α and iNOS in MG6 cells (Fig. 8a) and primary cultured microglia (Fig. 8b). A combined administration of TLR2 Ab and PAR2 siRNA further inhibited *P*. *gingivalis* infection-induced expression of IL-6, TNF-α and iNOS in MG6 cells (Fig. 8a) and primary cultured microglia (Fig. 8b).

Finally, a possible link between cell migration and inflammatory responses of microglia in response to *P*. *gingivalis* infection was examined by treatment with cytochalasin D, which can suppress cell migration by inhibiting actin polymerization. Treatment with cytochalasin D significantly inhibited the mRNA expression of proinflammatory mediators in microglia (Supplementary Fig. S4).

## Discussion

We herein show that the infection of microglial cells with *P*. *gingivalis* induced cell migration through secretion of gingipains and the subsequent proteolytic activation of PAR2. Furthermore, the combined use of KYT1 and KYT36 almost completely suppressed the *P*. *gingivalis* infection-induced cell migration and the PAR2 activation, suggesting that gingipains, Rgp and Kgp, are wholly responsible for these events. Although the potential involvement of extracellular nucleotides, including ADP or ATP, in the cell migration of microglia was widely known, neither P2Y_6_ receptors nor P2Y_12_ receptors was not involved in the *P*. *gingivalis* infection-induced microglial cell migration. Furthermore, the cell migration of MG6 cells was induced by LPS derived from *E*. *coli*, but not from *P*. *gingivalis*. These observations were consistent with a previous report to demonstrate that none of the TLR2-specific agonists stimulated migration in cultured microglia^28^. Therefore, it is considered that TLR4, but not TLR2, is involved in cell migration of microglia.

In our *in vivo* experiments, we utilized *CX3CR1*^+/*GFP*^ mice to evaluate microglial migration towards the injection site of *P*. *gingivalis*. It has been reported that brain-resident microglia expressed significantly higher level of *CX3CR1* than macrophages^29^. Furthermore, Yamasaki *et al*. have utilized *CX3CR1*^*+/GFP*^*CCR2*^*+/RFP*^ knock-in mice to show that CCR2 single-positive cells (infiltrating macrophages) exhibited elongated or spindle shape, whereas CX3CR1 single-positive cells (brain-resident microglia) showed a process-bearing morphology^30^. In the present study, most CX3CR1-positive cells that accumulated around the injected sites had a process-bearing morphology. Therefore, it is considered that brain-resident microglia could be delineated from infiltrating macrophages by counting process-bearing bright CX3CR1-positive cells. Furthermore, process-bearing bright CX3CR1-positive cells accumulated around the injection site were negative for Ki67, but only few CX3CR1-positive cells with spindle shape, presumably infiltrating macrophages, were positive for Ki67. These observations suggest that the accumulated cells around the injection site were mainly associated with cell migration rather than cell proliferation.

To address the intracellular signalings associated with PAR2 activation, we have focused on PI3K/Akt and MEK/ERK pathways because these two signaling pathways can induce microglial migration^31,32^. In the present study, infection of cultured microglia with *P*. *gingivalis* induced phosphorylation of Akt and ERK1/2. Our pharmacological studies showed that activation of PI3K/Akt and MEK/ERK pathways plays essential roles in the *P*. *gingivalis* infection-induced migration and membrane ruffling of microglia. The *P*. *gingivalis* infection-induced phosphorylation of Akt in microglia was decreased by the inhibition of PAR2. Furthermore, we provided evidence supporting the notion that Rgp and Kgp may regulate the cell migration of microglia through the proteolytic cleavage of PAR2 and the subsequent activation of the PI3K/Akt pathway. However, it was previously reported that Rgp and Kgp modulate neutrophil chemotaxis through the proteolytic cleavage of IL-8^33^. Furthermore, Rgp and Kgp promote monocyte migration through the proteolytic activation of matrix metalloprotease-9, which induces monocyte migration^34^. Therefore, the involvement of proteolytic cleavage other than the activation of PAR2 in the gingipain-mediated microglial migration cannot be totally ruled out, as the inhibition of PAR2-mediated signaling using either neutralizing Ab or siRNA significantly but not completely inhibited *P*. *gingivalis* infection-induced microglial migration.

In addition to the cell migration, the infection of microglia with *P*. *gingivalis* significantly increased the mRNA expression of proinflammatory mediators, including IL-6, TNF-α, and iNOS, without affecting the mRNA expression of anti-inflammatory mediators, including IL-10, arginase-1 and IL-4. The combined use of KYT1 and KYT36 significantly but incompletely inhibited the *P*. *gingivalis* infection-induced increased mRNA expression of proinflammatory mediators. These observations suggest that virulence factors other than gingipains, such as LPS and fimbriae, are also involved in the *P*. *gingivalis* infection-induced inflammatory responses. The infection of microglia with *P*. *gingivalis* also significantly increased the secretion of IL-6 and TNF-α, and the accumulation of NO metabolites. On the other hand, the combined administration of KYT1 and KYT36 significantly increased the extracellular levels of IL-6 and TNF-α, but significantly decreased NO metabolites, after infection of microglia with *P*. *gingivalis*. It has been reported that the cooperative action of Rgp and Kgp is also involved in the proteolytic degradation of various cytokines including TFN-α^35^ and IL-6^36,37^. Therefore, it is considered that Rgp and Kgp may limit the microglia-mediated neuroinflammation through proteolytic degradation of proinflammatory cytokines.

In the present study, the siRNA of PAR2 strongly inhibited the *P*. *gingivalis* infection-induced expression of proinflammatory mediators. A similar observation was reported by Uehara *et al*.^32^, who observed that Rgp and Kgp stimulate the secretion of proinflammatory cytokines by monocytes through the proteolytic cleavage of PAR-1, PAR-2 and PAR-3 with synergistic effects when used in combination with pathogen-associated molecular patterns. Furthermore, the inhibition of the PI3K/Akt pathway significantly inhibited the *P*. *gingivalis* infection-induced expression of IL-6, TNF-α and iNOS in microglia. Besides *P*. *gingivalis* LPS/TLR2 system, gingipain/PAR2 system was also involved in *P*. *gingivalis* infection-induced expression of inflammatory mediators by microglia. In the present study, TLR2 Ab and PAR siRNA had additive inhibitory effects, suggesting that infection of microglia with *P*. *gingivalis* activates gingipain/PAR2 system to induce neuroinflammation in a manner parallel to *P*. *gingivalis* LPS/TLR2 system. The precise mechanism by which microglia mediate chronic inflammation, which is responsible for the dementia associated with AD, remains unclear. One possible mechanism is that gingipain- and/or LPS-mediated acute neuroinflammation may induce the neuronal production of Aβ^5^, which may advance the acute neuroinflammation to the chronic state.

In this study, we have provided the first evidence that Rgp and Kgp cooperatively contribute to the cell migration of microglia towards the infected site and induction of neuroinflammation after infiltration into the brain. The cell migration associated with actin polymerization may be necessary for the subsequent inflammatory responses of microglia after activation of PAR2. Considering that Rgp and Kgp degrade components of the basal membrane, including type I collagen and fibronectin, and enhance the vascular permeability^38^, they may also contribute to the invasion of *P*. *gingivalis* into the brain (Supplementary Fig. S5). Therefore, our observations in this study may support “the infection hypothesis of Alzheimer’s disease”^1,9^.

## Methods

### Animals

*CX3CR1*^+/*GFP*^ mice on a C57/BL6J background (8–10 weeks old; Jackson Laboratory, Bar Harbor, ME, USA) were used. All animal experiments were conducted in accordance with the guidelines contained in the Act on Welfare and Management of Animals (Ministry of Environment of Japan) and Regulation of Laboratory Animals (Kyushu University) and under the protocols approved by the Institutional Animal Care and Use committee review panels at Kyushu University.

### Bacterial strains and culture conditions

*P*. *gingivalis* ATCC33277 and Lys gingipain mutant *P*. *gingivalis* KDP129 were used. *P*. *gingivalis* was maintained on blood agar plate containing 40 mg/ml trypto-soya agar (Nissui Pharmaceutical, Tokyo, Japan), 5 mg/ml brain heart infusion (Becton, Dickinson and Company, Franklin Lakes, NJ, USA), 1 g/ml cysteine (Wako Pure Chemical Industries, Osaka, Japan), 5 μg/ml hemin (Sigma-Aldrich, St. Louis, MO, USA), 1 μg/ml menadione (Sigma-Aldrich), 5% defibrinated sheep blood (Nippon Bio-test laboratories, Tokyo, Japan) in Bactron anaerobic chamber (Shel Lab, Cornelius, OR, USA) with 10% CO_2_, 10% H_2_, 80% N_2_^15^. KDP129 was maintained on blood agar plate with 20 μg/ml chloramphenicol (Wako Pure Chemical Industries). *P*. *gingivalis* was grown in enriched BHI broth containing 37 mg/ml brain heart infusion, 2.5 mg/ml yeast extract (Becton, Dickinson and Company), 1 g/ml cysteine, 5 μg/ml hemin and 1 μg/ml menadione. KDP129 was grown on BHI broth with 20 μg/ml chloramphenicol. Before *P*. *gingivalis* was cocultured with MG6 cells and primary cultured microglia, *P*. *gingivalis* culture medium was centrifuged (6000 × g, 10 min) and the supernatant was replaced with DMEM without FBS or penicillin-streptomycin.

### Microinjection and data acquisition

*CX3CR1*^+/*GFP*^ mice were anesthetized via an intraperitoneal injection of urethane and atropine (1.7 g/kg and 0.4 mg/kg, respectively) and positioned in a stereotaxic apparatus (Narishige, Tokyo, Japan), A cranial window was made in the somatosensory cortex, and the dura was carefully removed. A stab wound was made by inserting a 30-G needle into the somatosensory cortex (anterior, −1.7 mm; lateral, 2.0 mm, dorsoventral, 0.5 mm). Culture medium BHI, *P*.*gingivalis* in BHI or KDP129 in BHI (1 μl, 1 × 10^6^ CFU/ml) was injected. The flow rate of the injection was 10 μl/h maintained by a microsyringe pump (As One, Osaka, Japan), followed by waiting 10 min for total infiltration. Then skin was then sutured with 6.0-mm silk thread. The mice were killed 24 h after the injection.

### Migration assay *in vivo*

The microinjected mice were deeply anesthetized with an overdose of sodium pentobarbital (120 mg/kg, i.p.) and perfused transcardially with phosphate-buffered saline (PBS, pH 7.4) followed by 4% paraformaldehyde. The brains were obtained, cryoprotected for 2 days in 30% sucrose in PBS and then were embedded in an optimal cutting temperature compound (Sakura Finetechnical, Tokyo, Japan). Serial coronal frozen sections (14 μm) of the samples were prepared and mounted in Vectashield anti-fading medium (Vector Laboratories, Burlingame, CA, USA). Fluorescence images were taken using the confocal laser-scanning microscope (CLSM, 2si Confocal Laser-scanning Microscope; Nikon, Tokyo, Japan). All of the images were processed using the ImageJ 1.47 h software program (NIH). To account for any differences in the fluorescence intensity among the experiments, every image was set at the maximum threshold value (255), and all of the background was set to 0. To count the CX3CR1-GFP-positive cells, five squares (300 × 300 μm) were placed around the injection site of 3 independent sections per mouse, and the cells in those 5 squares were counted and statistically analyzed.

### MG6 cells

The *c-myc-*immortalized mouse microglial cell line MG6 (Riken Cell Bank, Ibaraki, Japan) was maintained in DMEM containing 10% fetal bovine serum (Invitrogen, San Diego, CA, USA) supplemented with 100 μM β-mercaptoethanol, 10 μg/ml insulin, 1% penicillin-streptomycin (Invitrogen), and 450 mg/ml glucose (Invitrogen).

### Primary cultured microglia

CD11b+ cells were isolated from the mouse brain by the MACS method. Twelve mice were anesthetized and transcardially perfused with PBS. The brains were obtained and cut into small pieces. After enzymatic digestion using a Neural Tissue Dissociation Kit (Miltenyi Biotec), the cell suspensions were further mechanically dissociated using a gentle MACS Dissociator (Milteny Biotec). The single-cell suspensions were obtained after being move to a 30mm cell strainer. After magnetic labeling with CD11b MicroBeads, the cell suspension was loaded onto a magnetic (MACS) column placed in the magnetic separator (Milteny Biotec). After rinsing the MACS column with PBS, the CD11b-positive fraction was collected according to previously described methods^39,40^.

### Cell migration assays *in vitro*

Cell migration was determined by using a Boyden chamber assay. A 12-well Boyden chamber (NeuroProbe, Gaithersburg, MD, USA) was used for the measurement of cell migration, in accordance with the manufacturer’s instruction. *E*. *coli* LPS, *P*. *gingivalis* LPS (Invivogen) and 6 × 10^5^ CFU/ml *P*. *gingivalis* in DMEM were placed into the lower wells, which were separated from the upper wells by polyvinylpyrrolidone-free polycarbonate filters (8-μm pore size, 25 × 80 mm; NeuroProbe). MG6 cells and primary cultured microglia were harvested by trypsinization, resuspended in DMEM, and added to the upper chamber at a density of 1.8 × 10^5^ cells/ml. Cells were incubated at 37 °C under cell culture conditions (95% air/5% CO_2_) for 12 h. At the end of the incubation, any non-migrating cells on the upper side of the membrane were removed with a cotton swab. Migrated cells on the lower part of the membrane were fixed in methanol for 10 min and stained with diff-quik (Sysmex, Kobe, Japan). Photomicrographs of randomly chosen fields were taken (BX-41; Olympus, Tokyo, Japan), and all cells were enumerated to calculate the average number of cells that had migrated.

### PAR2 knockdown with small interfering RNAs

MG6 cells and primary cultured microglia were seeded on 6-well plates at a density of 2 × 10^5^ cells/well in 2 ml of antibiotic-free DMEM. After 12 h, the cells were transiently transfected with control siRNA (sc-37007; Santa Cruz Biotechnology, Dallas, TX, USA), or PAR2 siRNA (sc-36187; Santa Cruz Biotechnology), using siRNA Transfection Reagent (sc-29528; Santa Cruz Biotechnology) in accordance with the manufacturer’s protocol. At 12 h after transfection, the cells were harvested for cell migration and RT-PCR.

### Immunofluorescent staining

Microinjection brain sections of *CX3CR1*^+/*GFP*^ mice were obtain for the proliferation assay, the samples were incubated with rabbit anti-Ki67 (SP6, 1:500; abcam) overnight at 4 °C, after being washed with PBS, they were incubated with donkey anti-rabbit Cy3 (1:500; Jackson ImmunoResearch, West Grove, PA, USA) for 2 h and then with Hoechst stain (1:200) and mounted in Vectashield anti-fading medium. Fluorescence images were taken using CLSM.

For the PAR2 activation assay, the infected MG6 cells and primary cultured microglia were fixed with 4% paraformaldehyde for 30 min. After washing with PBS, cells were then incubated with the goat anti-PAR2 (S-19, 1:1000; Santa Cruz Biotechnology) overnight at 4 °C. After being washed with PBS, they were incubated with donkey anti-goat Cy3 (1:500; Jackson ImmunoResearch, West Grove, PA, USA) for 2 h and then with Hoechst stain (1:200) and mounted in Vectashield anti-fading medium. Fluorescence images were taken using CLSM. An immunofluorescent intensity analysis of cleaved PAR2 staining was performed using the ImageJ 1.47 h software program (NIH).

For the actin polymerization assay, the infected MG6 cells were fixed with 4% paraformaldehyde for 30 min. After washing with PBS, cells were incubated with Texas Red-X Phalloidin (1 unit/well; Thermo Fisher Scientific, Waltham, MA, USA) for 1 h and then with Hoechst stain (1:200) and mounted in Vectashield anti-fading medium. Fluorescence images were taken using a CLSM. All of the images were processed using the ImageJ software program, and Z-stack images were projected along the Z-axis to recreate a two-dimensional (2D) representation of three-dimensional (3D) structures^39,40^.

### Immunoblotting analyses

The immunoblotting analyses were conducted as described previously^40^. In brief, each specimen was electrophoresed using 12% SDS-polyacrylamide gels. The proteins on the SDS gels were then electrophoretically transferred to nitrocellulose membranes. Following the blocking, the membranes were incubated at 4 °C overnight under gentle agitation with rabbit anti-Akt antibody and rabbit anti-pAkt (Ser 473) antibody (1:1000; Cell Signaling, Danvers, MA, USA), rabbit anti-ERK1/2 (1:1000; Cell Signaling Technology) and rabbit anti-pERK1/2 (1:1000; Cell Signaling Technology). After being washed, the membranes were incubated with horseradish peroxidase (HRP)-labeled anti-rabbit (1:1000; GE Healthcare, Tokyo, Japan) for 2 h at room temperature. Subsequently, the membrane-bound, HRP-labeled antibodies were detected using an enhanced chemiluminescence detection system (ECK lit, GE Healthcare, Tokyo, Japan) with an image analyzer (LAS-1000; Fuji Photo Film, Tokyo, Japan).

### Quantitative RT-PCR analysis

The mRNA isolated from MG6 cells and primary cultured microglia infected with *P*. *gingivalis* (MOI 1:5) at various time points were subjected to a quantitative RT-PCR. The total RNA was extracted with RNAiso Plus in accordance with the manufacturer’s instructions. A total of 800 ng of extracted RNA was reverse transcribed to cDNA using the QuantiTect Reverse Transcription Kit (Qiagen, Germantown, MD, USA). After an initial denaturation step at 95 °C for 5 m, temperature cycling was initiated. Each cycle consisted of denaturation at 95 °C for 5 s, annealing at 60 °C for 10 s, and elongation for 30 s. In total, 40 cycles were performed. The cDNA was amplified in duplicate using a Rotor-Gene SYBR Green RT-PCR Kit (Qiagen) with a Corbett Rotor-Gene RG-3000A Real-Time PCR system. The data were evaluated using the RG-3000A software program (version Rotor-Gene 6.1.93; Corbett). The sequences of primer pairs were described as follows: IL-6: 5′-TCAATTCCAG AAACCGCTATGA-3′ and 5′-CACCAGCATCAGTCCCAAGA-3′; iNOS: 5′-GCC ACCAACAATGGCAAC-3′ and 5′-CGTACCGGATG AGCTGTGAATT-3′; TNF-α: 5′-ATGGCCTCCCTCTCAGTTC-3′ and 5′-TTGGTGGTTTGCTACGACG TG-3′; IL-10: 5′-ATGCTGCCTGCTCTTACTGACTG-3′ and 5′-CCCAAGTAACCCTTA AAGTCCTGC-3′; Arginase-1: 5′-CGCCTTTCTCAAAAGG ACAG-3′ and 5′-CCA GCTCTTCATTGGCTTTC-3′; IL-4: 5′-TGGGTCTCAACCCCCAGCTAGT-3′ and 5′-TGCATGGCGTCCCTTCTCCTGT-3′. For data normalization, an endogenous control (actin) was assessed to control for the cDNA input, and the relative units were calculated by the comparative Ct method.

### Enzyme-linked immunosorbent assay (ELISA) and NO_2_^−^/NO_3_^−^ assay

The mean amounts of IL-6, TNF-α and NO_2_^−^ + NO_3_^−^ in the culture medium were measured after the infection of primary cultured microglia (2 × 10^6^ cell/well, 6-well plates) with *P*. *gingivalis* (MOI 1:5). IL-6 and TNF-α were measured by ELISA kits (R&D Systems) following the protocol provided by the manufacturer. The absorbance at 450 nm was determined using a microplate reader. NO_2_^-^ and NO_3_^-^ were measured by NO_2_^-^/NO_3_^-^ assay kits (Griess reagent kit; Dojindo, Tokyo, Japan) following the protocol provided by the manufacturer. The absorbance at 540 nm was determined using a microplate reader.

### Reagents

P2Y_12_ receptor inhibitor PSB0739 was obtained from Tocris Bioscience (Bristol, UK). P2Y_6_ receptor inhibitor MRS2578, MEK inhibitor U0126 and cytocharasin D were obtained from Sigma-Aldrich. PAR2 neutralization antibody (SAM11) was obtained from Santa Cruz Biotechnology, TLR2 neutralizing antibody from eBioscience (San Diego, CA, USA), and control Ab (mouse IgG2a) from BioLegend (San Diego, CA, USA). Rgp inhibitor KYT1 and Kgp inhibitor KYT36 were obtained from Peptide Institute (Osaka, Japan). PI3K inhibitor LY294002 was obtaind from EMD Milipore (Billerica, MA, USA), Akt inbibitor Akti from EMD Milipore. For *in vivo* studies, KYT1 and KYT36 were preincubated with *P*. *gingivalis* for 1 h. For *in vitro* studies, inhibitors and neutralizing enzymes were preincubated with MG6 cells and primary cultured microglia for 1 h.

### Statistical analysis

The data are represented as the mean ± standard error of the mean. The statistical analyses were performed using a two-tailed unpaired Student’s *t*-test and a one-way analysis of variance (ANOVA) with a post hoc Tukey’s test using the GraphPad Prism 7 Software package (GraphPad Software Inc., San Diego, CA, USA). A value of p < 0.05 was considered to indicate statistical significance.

## Electronic supplementary material

Supplementary Information

## References

[1] Itzhaki RF (2016). Microbes and Alzheimer’s diasease. J. Alzheimers Dis..

[2] Riviere GR, Riviere KH, Smith KS (2002). Molecular and immunologicale vidence of oral *Treponema* in the human brain and their association with Alzheimer’s disease. Oral Microbiol. Immunol..

[3] Poole S, Singhrao SK, Kesavalu L, Curtis MA, Crean S (2013). Determining the presence of periodontopathic virulence factors in short-term postmortem Alzheimer’s disease brain tissue. J. Alzheimers Dis..

[4] Pooles S (2015). Active invasion of Porphyromonas gingivalis and infection-induced component activation in ApoE-/- mice brain. J. Alzheimer’s Dis..

[5] Wu Z (2017). Cathepsin B plays a critical role in inducing Alzheimer’s Disease-like phenotypes following chronic systemic exposure to lipopolysaccharide from *Porphyromonas gingivalis* in mice. Brain Behav. Immunol..

[6] Itzhaki RF, Wozniak MA (2006). Herpess implex virus type1, apolipoprotein E, and cholesterol: a dangerous liaison in Alzheimer’s disease and other disorders. Prog. Lipid Res..

[7] Balin BJ (2008). Chlamydophila pneumonia and the etiology of late-onset Alzheimer’s disease. J. Alzheimers Dis..

[8] Miklossy J (2011). Alzheimer’s disease – a neurospinochetosis. Analysis of the evidence following Koch’s and Hill’s criteria. J. Neuroinflammation.

[9] Pisa D (2015). Different brain regions are infected with fungi in Alzheimer’s disease. Sci. Rep..

[10] Kumar DK (2016). Amyloid-β peptide protects against microbial infection in mouse and worm models of Alzheimer’s disease. Sci. Transl. Med..

[11] Olmos-Alonso A (2016). Pharmacological targeting of CSF1R inhibits microglial proliferation and prevents the progression of Alzheimer’s-like pathology. Brain.

[12] Spangenberg EE (2016). Eliminating microglia in Alzheimer’s mice prevents neuronal loss without modulating amyloid-β pathology. Brain.

[13] Ide M (2016). Periodontitis and cognitive decline in Alzheimer’s disease. PLoS One.

[14] Liu Y (2013). Leptomeningeal cells transduce peripheral macrophage inflammatory signal to microglia in response to Porphyromonas gingivalis LPS. Med. Inflamm..

[15] Takayama F, Hayashu Y, Wu Z, Liu Y, Nakanishi H (2016). Diurnal dynamic behavior of microglia in response to infected bacteria through the UDP-P2Y_6_ receptor system. Sci. Rep..

[16] Potempa J, Pike R, Travis J (1995). The multiple forms of tryprin-like activity present in various strains of *Porphyromonas gingivalis* are due to the presence of either Arg-gingipain or Lys-gingipain. Infect. Immun..

[17] Kadowaki T (2000). *Porphyromonas gingivalis* proteinases are virulenve determinants in progression of priodontal diseases. J. Biochem..

[18] Kadowaki T (2007). A role for gingipains in cellular responses and bacterial survival in *Porphyromonas gingivalis*-infected cells. Front Biosci..

[19] Ransohoff RM (2009). Chemokines and chemokine receptors: standing at the crossroads of immunobiology and neurobiology. Immunity.

[20] Lourbakos A (2001). Arginine-specific protease from Porphyromonas gingivalis acyivates protease-activated receptors on human oral epithelial cells and induces interleikin-6 secretion. Infect. Immun..

[21] Holzhausen M, Spolidorio LC, Vergnoile N (2006). Protease-activated receptor-2 activation: a major role in the pathogenesis of *Porphyromonas gingivalis* infection. Am. J. Pathol..

[22] Giacaman RA, Asrani AC, Ross KF, Herzberg MC (2009). Cleavage of protease-activated receptors on an immortalized oral epithelial cell line by Porphorymonas gingivalis gingipains. Microbiology.

[23] Ishida Y, Nagai A, Kobayashi S, Kim SU (2006). Upregulation of protease- activated receptor-1 in astrocytes in Parkinson Disease: astrocyte-mediated neuroprotection through increased levels of glutathione peroxidase. J. Neuropathol. Exp. Neurol..

[24] Lee NR (2016). House dust mite allergen suppresses neutrophil apoptosis by cytokine release via PAR2 in normal and allergic lymphocytes. Immunol. Res..

[25] Kanke T (2001). Protease-activated receptor-2-mediated activation of stress-activated protein kinases and inhibitory κB kinase in NCTC 2544 keratinocytes. J. Biol. Chem..

[26] Rothmeier AS, Ruf W (2012). Protease-activated receptor 2 signaling in inflammation. Semin. Immunopathol..

[27] Paez J, Sellers WR (2003). PI3K/PTEN/AKT pathway. A critical mediator of oncogenic signaling. Cancer Treat. Res..

[28] Vinnakota K (2013). Toll-like receptor 2 mediates microglia/brain macrophage MT1-MMP expression and glioma expansion. Neuro. Oncol..

[29] Hickmann SE (2013). The microglial sensome revealed by direct RNE sequencing. Nat. Neurosci..

[30] Yamasaki R (2014). Differential roles of microglia and monocytes in the inflammaed central nervous system. J. Exp. Med..

[31] Ohsawa K (2007). Involvement of P2X_4_ and P2Y_12_ receptors in ATP-induced microglial chamotaxisis. Glia.

[32] Irino Y, Nakamura Y, Inoue K, Kohsaka S, Ohsawa K (2008). Akt activation is involved in P2Y_12_ receptor-mediated chemotaxis of microglia. J. Neurosci. Res..

[33] Uehara A, Imamura T, Potempa J, Travis J, Takada H (2008). Gingipains of *Porphyromonas gingivalis* synergistically induce the production of proinflammatory cytokines through protease-activated receptors with Toll-like receptor and NOD1/2 ligands in human monocytic cells. Cell Microbiol..

[34] Zhou J, Zhang J, Chao J (2012). *Porphorymonas gingivalis* promotes monocyte migration by activating MMP-9. J. Periodont. Res..

[35] Calkins CC, Platt K, Potempa J, Travis J (1998). Inactivation of tumor necrosis factor-α by proteinases (gingipains) from the periodontal paythogen, *Porphyromonas gingivalis*. Implication of immune evasion. J. Biol. Chem..

[36] Steffen MJ, Holt SC, Ebersole JL (2000). *Prophyromonas gingivalis* induction of mediators and cytokine secretion by human gingival fibroblasts. Oral Microbiol. Immunol..

[37] Bodet C, Chandad F, Grenier D (2005). Modulation of cytokine production by *Porphyromonas gingivalis* in a macrophages and epithelial cell co-culture model. Microbes. Infect..

[38] Kadowaki T (2004). Suppression of pathogenicity of *Porphyromonas gingivalis* by newly developed gingipain inhibitors. Mol. Pharmacol..

[39] Zhang X, Wu Z, Hayashi Y, Okada R, Nakanishi H (2014). Peripheral role of cathepsin S in Th1 cell-dependent transition of nerve injury-induced acute pain to a chronic state. J. Neurosci..

[40] Ni J (2015). Critical role of proteolytic relay through cathepsins B and E in the phenotypic change of microglia/macrophage. J. Neurosci..

