# Author Correction: Infection of microglia with *Porphyromonas gingivalis* promotes cell migration and an inflammatory response through the gingipain-mediated activation of protease-activated receptor-2 in mice

**DOI:** 10.1038/s41598-018-27508-9

**Published:** 2018-07-04

**Authors:** Yicong Liu, Zhou Wu, Yurika Nakanishi, Junjun Ni, Yoshinori Hayashi, Fumiko Takayama, Yanmin Zhou, Tomoko Kadowaki, Hiroshi Nakanishi

**Affiliations:** 1 Department of Aging Science and Pharmacology, Faculty of Dental Sciences, Kyushu University, Fukuoka, 812-8582 Japan; 2 OBT Research Center, Faculty of Dental Sciences, Kyushu University, Fukuoka, 812-8582 Japan; 3 Department of Implantology, School of Stomatology, Jilin University, Changchun, 130021 China; 4 Division of Frontier Life Science, Department of Medical and Dental Sciences, Graduate School of Biomedical Sciences, Nagasaki University, Nagasaki, 852-8588 Japan

Correction to: *Scientific Reports* 10.1038/s41598-017-12173-1, published online 18 September 2017

The original version of this Article contained a typographical error in the spelling of the author Tomoko Kadowaki, which was incorrectly given as Tomoko Kadawaki. This has now been corrected in the PDF and HTML versions of the Article, and the Supplementary Information file that accompanies the Article.

