# Supplementary material for: Infection of microglia with Porphyromonas gingivalis promotes cell migration and an inflammatory response through the gingipain-mediated activation of protease-activated receptor-2 in mice
Source: Sci Rep. 2017 Sep 18;7:11759. doi: 10.1038/s41598-017-12173-1 (PMC5603557; doi:10.1038/s41598-017-12173-1)
Supplement: Supplementary file 1 — Supplementary Information [file 41598_2017_12173_MOESM1_ESM.pdf]

Supplementary Information for

Infection of microglia with *Porphyromonas gingivalis* promotes cell migration and an inflammatory response through the gingipain-mediated activation of protease-activated receptor-2 in mice

Yicong Liu[1], Zhou Wu[1,2], Yurika Nakanishi[1], Junjun Ni[1], Yoshinori Hayashi[1], Fumiko Takayama[1], Yamin Zhou[3], Tomoko Kadowaki[4], Hiroshi Nakanishi[1,*]

[1]Department of Aging Science and Pharmacology, [2]OBT Research Center, Faculty of Dental Sciences, Kyushu University, Fukuoka 812-8582, Japan. [3]Department of Implantology, School of Stomatology, Jilin University, Changchun 130021, China. [4]Division of Frontier Life Science, Department of Medical and Dental Sciences, Graduate School of Biomedical Sciences, Nagasaki University, Nagasaki 852-8588, Japan

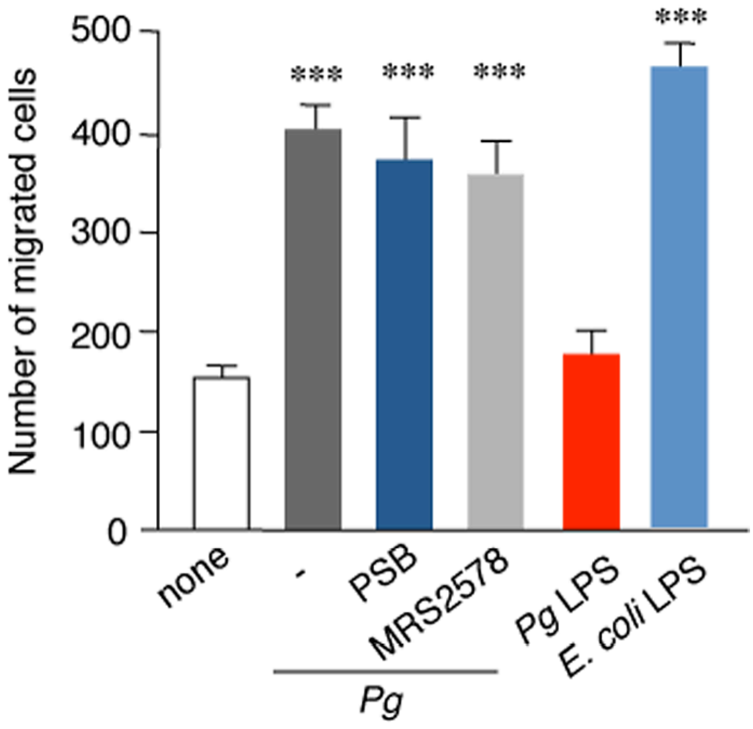

Supplementary Figure S1. Microglial migration induced by infection with *P. gingivalis* in the absence or presence of PSB or MRS2578 and treatment with LPS derived from *P. gingivalis* or *E. coli*. The quantitative analyses of the number of cells that migrated after the infection of MG6 cells with *P. gingivalis* in the absence or presence of PSB (1 μM) or MRS2578 (1 μM) and treatment with LPS (1 μg/ml) or *E. coli* LPS (100 ng/ml). MG6 cells that migrated through a membrane were stained and counted after 12 h. The columns and bars represent the mean ± SEM (n=3). A one-way ANOVA with post hoc Tukey's test; none vs. *Pg*: $^{***}p$=0.0001, *Pg* vs. *Pg*+PSB: p=0.9821, *Pg* vs. *Pg*+MRS2578: p=0.9629, none vs. *Pg* LPS: p=0.5006, none vs. *E. coli* LPS: $^{***}p$=0.0001.

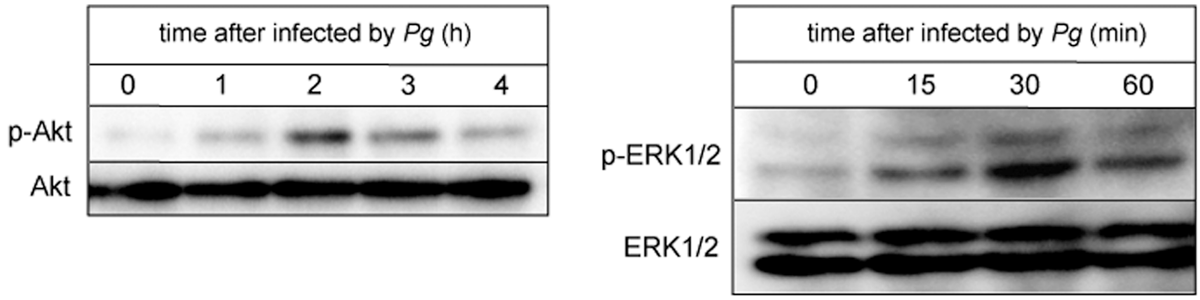

Supplementary Figure S2. PAR2 activation and subsequent phosphorylation of Akt and ERK1/2 after infection of microglia with *P. gingivalis*. The immunoblots show phosphorylation of Akt and ERK1/2 after infection of MG6 cells (2×10^6 cell/well, 6 -well plates) with *P. gingivalis* (MOI 1:5) at each time point.

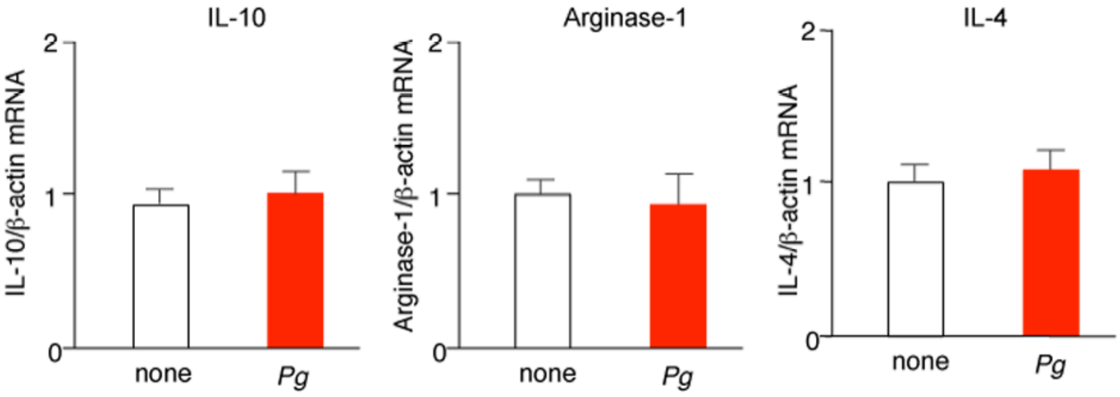

Supplementary Figure S3. Effects of *P. gingivalis* infection on the expression of anti-inflammatory mediators in microglia. The quantitative analyses of the mRNA expression of IL-10, Arginase-1 and IL-4 after the infection of MG6 cells (2×10^6 cell/well, 6 well plate) with *P. gingivalis* (MOI 1:5) at 12h. The columns and bars represent the mean±SEM (n=3). A two-tailed unpaired t-test; IL-10 group: p=0.4894. Arginase-1 group: p=0.569. IL-4 group: p=0.6172.

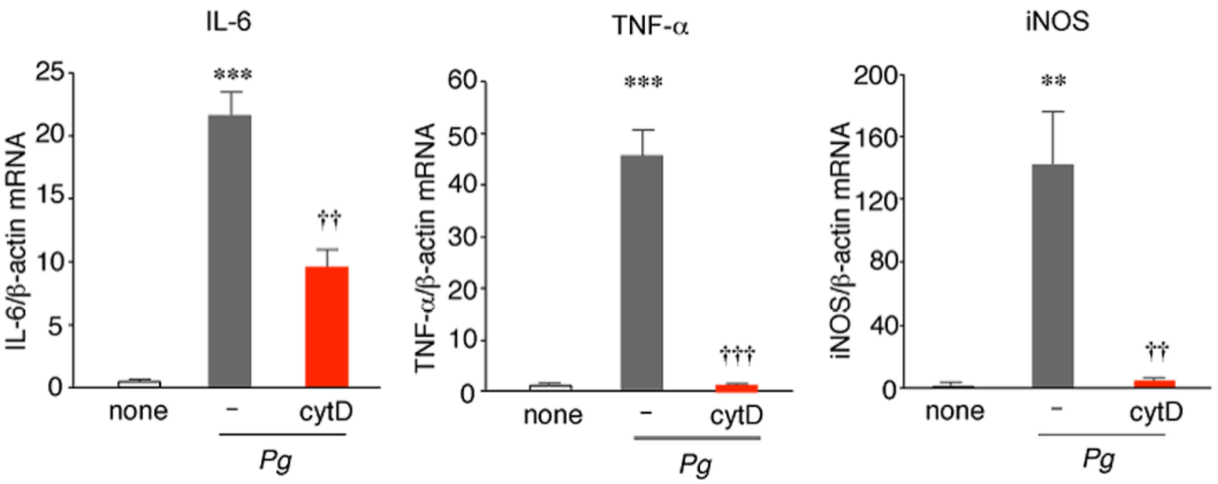

Supplementary Figure S4. A possible link between cell migration and inflammatory responses of microglia in response to *P. gingivalis* infection. The quantitative analyses of the mRNA expression of IL-6 , TNF-α and iNOS after the infection of MG6 cells (2×10^6 cell/well, 6-well plates) with *P. gingivalis* (MOI 1:5) in the presence and absence of cytochalasin D (cytD, 1 μM) at 12 h. The columns and bars represent the mean±SEM (*n*=3). A one-way ANOVA with post hoc Tukey's test; none vs. *Pg, Pg* vs. *Pg*+cytD were as follows: IL-6 group: $^{***}p$=0.0003, $^{††}p$=0.0042. TNF-α group: $^{***}p$=0.0001, $^{†††}p$=0.0001, iNOS group: $^{**}p$=0.0047, $^{††}p$=0.0047

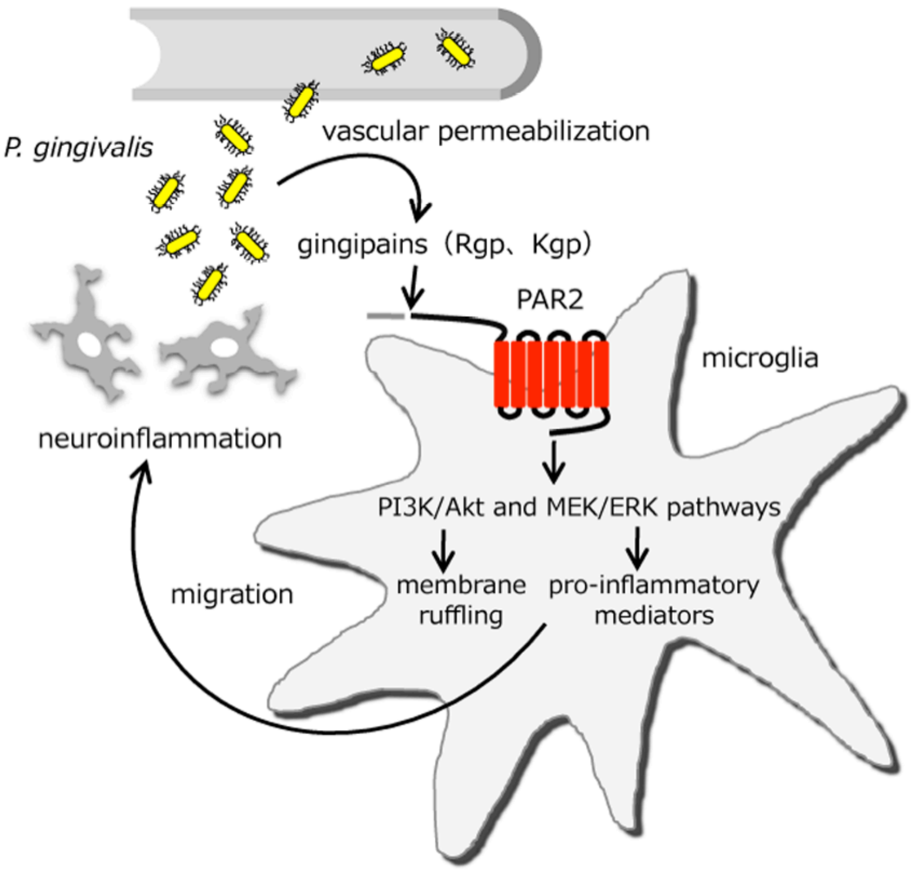

Supplementary Figure S5. A schematic diagram of cellular activation of microglia after infection of *P. gingivalis*. Gingipains (Rgp and Kgp) secereted from infected *P. gingivalis* activates PI3K/Akt and MEK/ERK pathways through proteolytic activation of PAR2, leading to induce migratory and inflammatory responses of microglia.

For Figure 4c

p-Akt

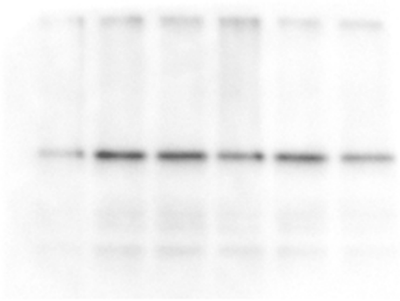

Akt

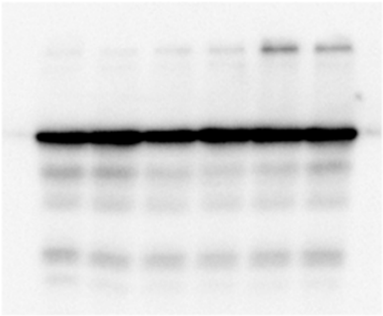

For Figure 4e

p-ERK1/2

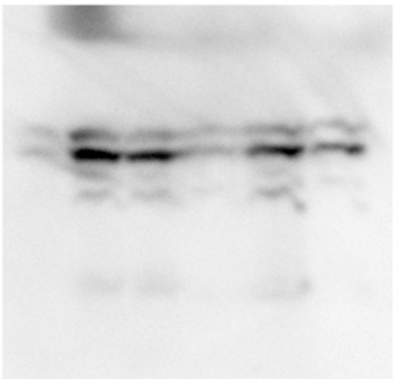

ERK1/2

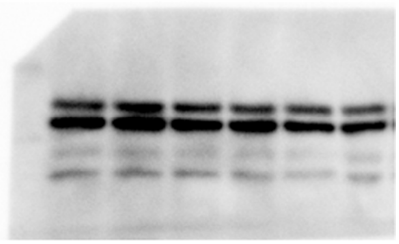

Supplementary Figure S6. Full-length blots used for Figure 4.
